# Autoimmune Epilepsy Secondary to CASPR2 Antibody Encephalitis: A Case Report Highlighting Diagnostic Challenges and Management

**DOI:** 10.7759/cureus.93844

**Published:** 2025-10-04

**Authors:** Fenghui Ye, Nikita Srinivasan, Joanna Suski, Mehdi Ghasemi

**Affiliations:** 1 Neurology, Tufts Medical Center, Boston, USA; 2 Neurology, Lahey Hospital and Medical Center, Burlington, USA

**Keywords:** autoimmune encephalitis, caspr2, dysautonomia, epilepsy, seizure

## Abstract

CASPR2 (contactin-associated protein-like 2) is a cell adhesion molecule expressed in both the central and peripheral nervous systems. CASPR2 autoantibodies are associated with autoimmune encephalitis and epilepsy; however, the initial presentation and subsequent treatment can be challenging. We report a rare case of CASPR2-related new-onset seizures, culminating in a motor vehicle accident, as well as dysautonomia in a 50-year-old man with a prior history of vasovagal syncope. The patient experienced three types of seizures: (i) focal preserved consciousness seizures, (ii) focal preserved consciousness seizures/dysautonomia, and (iii) focal impaired consciousness seizures. Post-ictal confusion included stripping clothes off due to feeling hot. Video electroencephalogram (EEG) monitoring confirmed bilateral temporal lobe seizures. Further evaluation revealed anti-CASPR2 antibody positivity in both serum and cerebrospinal fluid (CSF), consistent with autoimmune epilepsy. Despite treatment with intravenous (IV) methylprednisolone (1 g/day for five days) and multiple antiseizure medications (levetiracetam, lacosamide, and oxcarbazepine), the patient experienced persistent recurrent seizures, cognitive deficits, and medication-related side effects. This prompted adding IV immunoglobulin therapy, which helped with seizure control. This case underscores the diagnostic challenges of CASPR2 autoimmune epilepsy and highlights the importance of early recognition and treatment.

## Introduction

Autoimmune encephalitis encompasses a group of inflammatory brain disorders driven by the immune system's attack on self-antigens within the central nervous system (CNS). These conditions are increasingly recognized as causes of acute neuropsychiatric syndromes, especially in patients with seizures, cognitive decline, and behavioral changes [1]. Among the identified autoantibodies, those targeting contactin-associated protein-like 2 (CASPR2) have emerged as clinically significant, offering insights into diagnosis and immunotherapy [2].

CASPR2, encoded by the *CNTNAP2* gene, is a transmembrane protein of the neurexin superfamily, localized at juxtaparanodal regions of myelinated axons where it regulates voltage-gated potassium channels (Kv1.1 and Kv1.2) clustering and neuronal excitability [2]. Its expression in both the CNS and the peripheral nervous system (PNS) links it to a range of disorders, including epilepsy, autism spectrum disorder, neuromyotonia, and Morvan syndrome [3-6]. CASPR2 autoantibodies are most commonly associated with limbic encephalitis, presenting with memory impairment, mood disturbances, and seizures, sometimes including faciobrachial dystonic seizures (FBDS) [7-9].

Diagnosis involves clinical assessment, neuroimaging, cerebrospinal fluid (CSF) analysis, and antibody testing, with cell-based assays being the gold standard [10,11]. CASPR2 antibodies in both serum and CSF correlate with more severe disease [10]. This case report aims to provide a detailed account of a patient with anti-CASPR2 antibody-related autoimmune seizures, illustrating the diagnostic journey, therapeutic interventions, and clinical outcomes. By presenting this case, we seek to contribute to the growing body of literature on autoimmune encephalitis and advocate for the inclusion of CASPR2 antibody testing in the differential diagnosis of patients with unexplained seizures, cognitive changes, or other neuropsychiatric symptoms. Enhanced recognition of CASPR2-associated syndromes may facilitate earlier intervention, improve patient outcomes, and deepen our understanding of the immunopathogenesis underlying these complex disorders.

## Case presentation

A 50-year-old male construction company owner with a long-standing history of vasovagal syncope, averaging one episode annually, presented with new-onset spells of dizziness, staring, and unresponsiveness beginning in January 2024. Initially misdiagnosed as syncope, these episodes progressively increased in frequency, culminating in a motor vehicle accident on November 25, 2024. During this accident, the patient swerved across four lanes of traffic, striking another vehicle. Although airbags deployed, it remains uncertain whether he sustained a head injury. The patient has no recollection of exiting the vehicle and was found by EMS walking alongside the highway. He was admitted to the hospital from November 25 to November 28, 2024. While in the emergency department, he experienced a seizure characterized by staring, unresponsiveness, and nonsensical speech lasting two minutes, without abnormal jerking movements.

The patient's past medical history includes vasovagal syncope since age 10, with no prior history of head trauma, meningitis, or encephalitis. Family history is notable for malignancy, with a paternal first cousin and maternal aunt succumbing to cancer; however, no epilepsy or autoimmune disorders have been reported. Social history reveals he is a former smoker, having quit over 20 years ago, and he has no history of alcohol abuse. The initial diagnostic workup, summarized in Table 1, was unremarkable and included a complete blood count (CBC), basic metabolic panel (BMP), liver function tests (LFTs), lipase, lactic acid, an alcohol/drug screen, a non-contrast head CT scan, and a head/neck CT angiography. Additionally, the brain MRI, both with and without contrast, during hospitalization, was unremarkable.

**Table 1:** The results of blood testing in the presented case with autoimmune epilepsy secondary to CASPR2 antibody encephalitis. *Autoimmune epilepsy antibody panel includes CASPR2 (contactin-associated protein-like 2), GAD65 (glutamic acid decarboxylase 65), VGKC (voltage-gated potassium channel), LGI1 (leucine-rich glioma-inactivated 1), and NR1 (NR1 subunit of the N-methyl-D-aspartate (NMDA) receptor) antibodies. WBC: white blood cells; RBC: red blood cells; BUN: blood urea nitrogen; AST: aspartate aminotransferase; ALT: alanine aminotransferase.

Measurements	Results			Reference Range
	November 25, 2024	December 19, 2024	January 08, 2025	
WBC count	6.74	-	-	4.00–10.8 K/uL
RBC count	6.11	-	-	4.50–5.90 M/uL
Platelet count	205	-	-	150–400 K/uL
Sodium	139	-	137	134–146 mmol/L
Potassium	4.4	-	4.1	3.4–5.2 mmol/L
Chloride	105	-	105	98–107 mmol/L
BUN	14	-	17	7–24 mg/dL
Creatinine	1.11	-	1.01	0.5–1.30 mg/dL
Glucose	110	-	100	70–118 mg/dL
Calcium	9.3	-	9.3	8.3–10.6 mg/dL
AST	13	-	13	11–40 U/L
ALT	14	-	19	7–40 U/L
Lipase	39	-	-	0–68 U/L
Lactic acid	0.9	-	-	0.5–2.0 mmol/L
Alcohol level	<10	-	-	<10 mg/dL
Acetaminophen level	<3	-	-	<30 µg/mL
Salicylate level	<5	-	-	<30 mg/dL
Autoimmune epilepsy antibody panel*	-	Positive anti-CASPR2 antibody	-	Negative
Neuromyelitis optica/AQP4 IgG antibody	-	-	Negative (<1:10)	Negative (<1:10)
Myelin oligodendrocyte glycoprotein (MOG) IgG antibody	-	-	Negative (<1:10)	Negative (<1:10)
Anti-thyroid peroxidase antibody	-	-	Negative (<3 IU/mL)	Negative (<78 IU/mL)
Epstein-Barr virus IgM antibody	-	-	Negative	Negative

**Figure 1:**
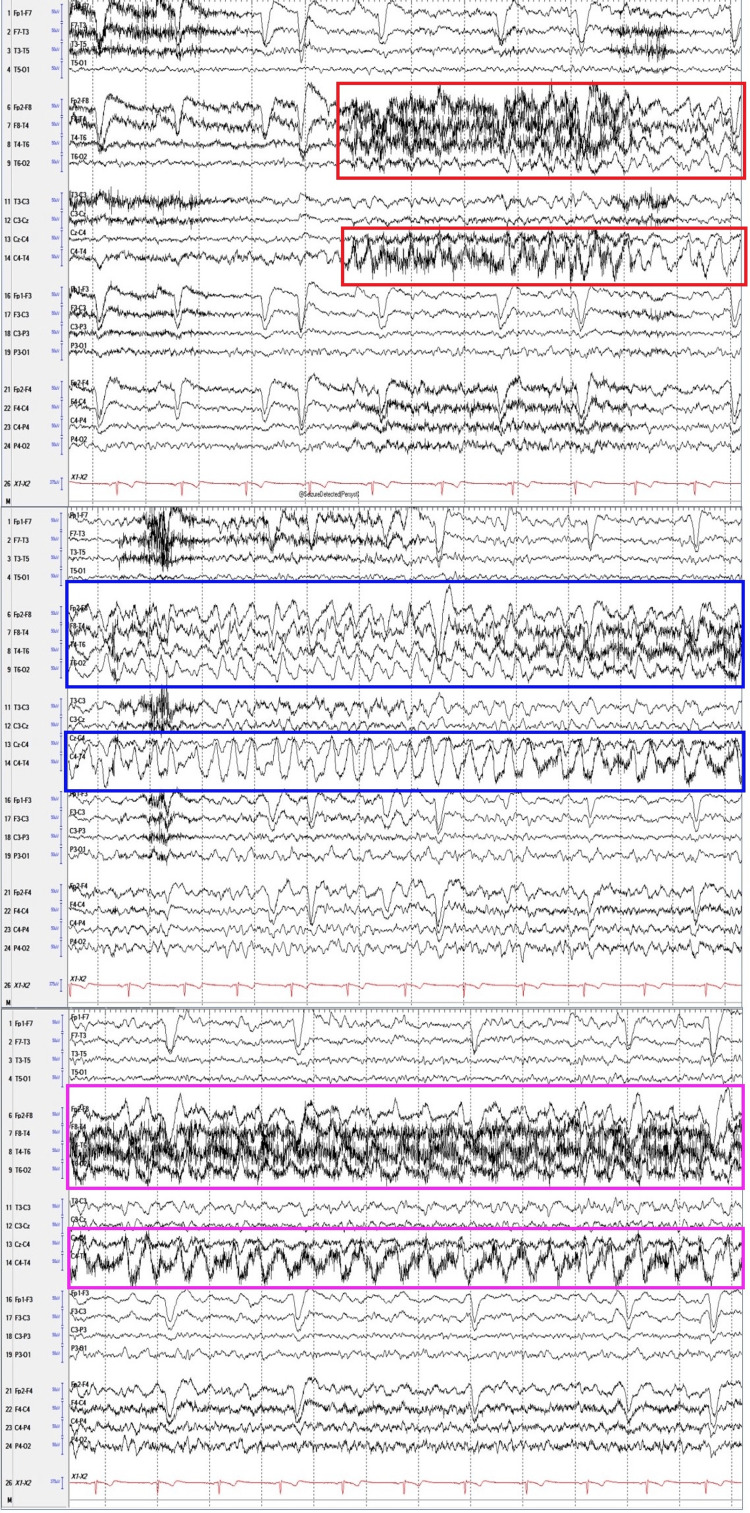
Long-term video electroencephalogram (vEEG) using a temporal-central parasagittal montage revealed rhythmic 2–3 Hz activity maximal at the T4 region, which evolved into higher amplitude, slowed to 1 Hz, and abruptly ceased after 40 seconds. Red-line box: Seizure onset marked by rhythmic 3 Hz activity, most prominent at T4 and best visualized in the parasagittal chains. Blue-line box: Morphology changes with increased amplitude and sharply contoured waveforms. Purple-line box: Frequency decreases to 1.5–2 Hz.

Given suspicion of seizure activity, levetiracetam 750 mg orally twice daily was initiated during hospitalization. Long-term EEG monitoring revealed focal epileptic seizures characterized by rhythmic 2-3 Hz activity at the T4 region, evolving into higher amplitude, slowing to 1 Hz, and abruptly ceasing after 40 seconds (Figure 1). Consequently, levetiracetam was increased to 1000 mg orally twice daily. Subsequent 24-hour EEG monitoring showed no electrographic seizures, and the patient was discharged on this regimen with follow-up scheduled at the epilepsy clinic.

During his follow-up visit on December 19, 2024, the patient reported three distinct seizure types: (i) Focal preserved consciousness seizures occurred three to four times daily, characterized by déjà vu, sweating, dizziness, and olfactory hallucinations (*e.g.*, coffee scent), lasting 15-20 seconds. Notably, sweating and dizziness could also represent symptoms of dysautonomia in this case; (ii) focal preserved consciousness seizures/dysautonomia manifested as a numb feeling in the body that comes in waves, goosebumps, and light-headedness, occurring multiple times per day; and (iii) focal impaired consciousness seizures, occurring three to four times weekly, were typified by staring, unresponsiveness, nonsensical speech, and postictal confusion lasting several minutes. The patient also reported significant cognitive decline, including difficulties with work-related tasks, short-term memory deficits, challenges with facial recognition, trouble forming sentences, and difficulty understanding conversations. Additional symptoms included emotional lability, irritability, and insomnia. Vasovagal episodes, triggered by emotional stress (*e.g.*, seeing blood), manifested as sweating, pallor, and transient loss of consciousness without postictal confusion. Generalized muscle twitching, involving the shoulders, glutes, and face, was reported but visually unconfirmed. Subsequently, lacosamide therapy was initiated at 50 mg orally twice daily, titrated to 150 mg twice daily.

The constellation of symptoms raised concern for an autoimmune or paraneoplastic process. Therefore, serum autoimmune encephalitis/paraneoplastic antibody panel testing was performed and revealed anti-CASPR2 antibody positivity (serum titer: 1:100), warranting hospital readmission from January 8 to 10, 2025, for further expedited management. Lumbar puncture results showed a protein level of 29 mg/dL, a glucose level of 75 mg/dL, and a CSF cell count of 4 cells/µL, with 95% lymphocytes (Table 2). Herpes Simplex Virus PCR testing was negative. The autoimmune antibody panel confirmed anti-CASPR2 antibody positivity (CSF titer: 1:160).

**Table 2:** Cerebrospinal fluid (CSF) study in the presented case with autoimmune epilepsy secondary to CASPR2 antibody encephalitis. * The encephalitis/paraneoplastic autoantibody panel includes ANNA1 (HU), ANNA2 (RI), ANNA3, PCA1 (YO), PCA2, PCA TR (DNER), AGNA/SOX1 Glial nuclear type 1, Amphiphysin, CRMP5/CV2, GAD65, MA2/TA, Myelin, AQP4, NMDAR1, AMPAR1, AMPAR2, GABABR, LGI1, CASPR2, DPPX Receptor, and VGKC antibodies.

Measurements (January 09, 2025)	Results	Reference Range
WBC count	4	≤10 cells/uL
RBC count	<500	<500 cells/uL
Protein	29	15–45 mg/dL
Glucose	75	40–70 mg/dL
CSF culture	No growth after 3 days	No growth after 3 days
Cytomegalovirus by PCR	Not detected	Not detected
Herpes simplex virus by PCR	Not detected	Not detected
Encephalitis/paraneoplastic autoantibody panel*	Positive anti-CASPR2 Ab (Titer >1:160)	Negative (Titer <1:10)
CSF cytology	No malignant cells	No malignant cells
CSF flow cytometry	No abnormal lymphocyte population	No abnormal lymphocyte population

EEG monitoring during sleep revealed rhythmic activity buildup in the left temporal region, evolving over several seconds and lasting 30 seconds, without clinical correlation and suggestive of subclinical seizures. CT imaging of the chest, abdomen, and pelvis with contrast was negative for malignancy. The patient was treated with IV methylprednisolone 1 g daily for five days and discharged on January 10, 2025, with levetiracetam 1000 mg twice daily and lacosamide 200 mg twice daily. Further steroid therapy and consideration of other potential immunotherapy agents, such as IVIG, were deferred until his follow-up visit within three weeks of discharge.

Post-discharge, the patient remained seizure-free for approximately one month but later experienced breakthrough seizures, including episodes of confusion, automatisms, and postictal symptoms, such as unresponsiveness with lip smacking during a phone call. Oxcarbazepine 600 mg orally twice daily was added for seizure control. Despite treatment, cognitive deficits, emotional lability, and irritability persisted. The patient continued to experience generalized muscle twitching. To evaluate for CASPR2 antibody-associated peripheral nerve hyperexcitability syndrome, an electromyography (EMG) and nerve conduction study (NCS) were performed, with attention to findings such as increased insertional activity, myokymic discharges, fasciculations, and neuromyotonic discharges. However, the results were unremarkable, showing no evidence of large-fiber peripheral neuropathy, hyperexcitability, polyradiculopathy, or myopathy (Tables 3, 4).

**Table 3:** Nerve conduction studies (NCS) in the presented case with autoimmune epilepsy secondary to CASPR2 antibody encephalitis. ADM: abductor digiti minimi; AHB: abductor hallucis; APB: abductor pollicis brevis; EDB: extensor digitorum brevis; NL: normal.

Site	Latency (ms)		Amplitude (Motor=mV; Sensory=µV)		Conduction Velocity (m/s)	
	Patient's value	NL range	Patient’s value	NL range	Patient's value	NL range
Right peroneal (EDB) motor						
Ankle	4.2	<6.5	6.5	>1.10	-	-
Below the fibular head	11.8	-	6.1	-	46	>36
Lateral popliteal fossa	13.3	-	6.1	-	67	≥42
Right tibial (AHB) motor						
Ankle	4.6	<6.1	8.5	>5.3	-	-
Knee	13.4	-	6.7	-	49	≥42
Right median (APB) motor						
Wrist	4.1	<4.7	13.2	>4.2	-	-
Elbow	9	-	12.1	-	55	>47
Right ulnar (ADM) motor						
Wrist	3.1	<3.7	13.7	>5.9	-	
Below elbow	7.7	-	12.5	-	54	>52
Above elbow	9.6	-	12.4	-	53	>43
Right radial sensory						
Forearm-wrist	2.4	<2.8	26	>7	59	≥40
Right sural sensory						
Calf-lateral malleolus	3.1	<4.5	16	>4	58	≥40

**Table 4:** Electromyography (EMG) in the presented case with autoimmune epilepsy secondary to CASPR2 antibody encephalitis. Fasc: fasciculation potentials; FDI: first dorsal interosseous; Fibs: fibrillation potentials; NL: normal; Poly: polyphasic; PSW: positive sharp waves; Recrt: recruitment; Spon Disc: spontaneous discharges.

Muscle (All Right Side)	Ins Act	Fibs/PSW	Fasc	Spon Disc	Amplitude	Duration	Poly	Recrt	Activation
Medial gastrocnemius	NL	NL	NL	NL	NL	NL	NL	NL	NL
Tibialis anterior	NL	NL	NL	NL	NL	NL	NL	NL	NL
Vastus medialis	NL	NL	NL	NL	NL	NL	NL	NL	NL
FDI	NL	NL	NL	NL	NL	NL	NL	NL	NL
Pronator teres	NL	NL	NL	NL	NL	NL	NL	NL	NL
Triceps	NL	NL	NL	NL	NL	NL	NL	NL	NL
Biceps	NL	NL	NL	NL	NL	NL	NL	NL	NL
Deltoid	NL	NL	NL	NL	NL	NL	NL	NL	NL

The follow-up plan included a 12-week course of IV methylprednisolone (1 g weekly for six weeks, starting at the end of January 2025, then biweekly for six weeks) and IVIG therapy (a total of 2 g/kg administered over three days from March 12 to 14, 2025). Overall, this immunotherapy strategy was selected based on emerging evidence and consensus guidelines for CASPR2 antibody-associated autoimmune encephalitis [12,13]. Subsequently, IVIG was planned at 0.5 g/kg every six weeks, with continued methylprednisolone therapy for seven additional weeks. Adjustments were based on therapeutic response. Antiseizure medication doses were modified to levetiracetam 1500 mg twice daily, lacosamide 200 mg twice daily, and oxcarbazepine 300 mg in the morning and 600 mg in the evening. These changes resulted in seizure freedom during the follow-up visit in May 2025.

Additionally, the patient underwent formal neuropsychological testing in late May 2025, which revealed intact baseline intellectual abilities and generally strong performance across most cognitive domains, including basic attention and working memory, executive functions (including cognitive flexibility, inhibition, and planning), confrontation and auditory naming, verbal fluency, and visuospatial skills. However, he showed broadly reduced encoding on memory tasks, with particular difficulty in retention and retrieval on verbal memory measures. Semantic fluency was notably weaker (average) compared to his high performance on phonemic fluency, and he exhibited an elevated number of repetition errors across verbal fluency trials. Overall, these deficits in verbal learning and memory, along with subtle vulnerability in semantic access efficiency, mainly suggest dysfunction in left temporal systems. Accordingly, the patient started cognitive rehabilitation therapy (to learn new compensatory strategies, particularly those aimed at supporting verbal learning and memory).

## Discussion

This case highlights the diagnostic challenges in distinguishing autoimmune epilepsy from other etiologies of seizures, particularly in patients presenting with a history of transient loss of consciousness or syncope. The identification of CASPR2 antibodies in both CSF and serum supported the diagnosis of autoimmune epilepsy. Notably, CASPR2 antibodies are more reliably identified in serum than in CSF, particularly in patients with peripheral nerve hyperexcitability syndromes [9,14,15]. While CSF positivity is highly specific for CNS involvement, such as limbic encephalitis, seizures, and cognitive impairment, serum testing offers greater sensitivity and should be prioritized in diagnostic evaluation [9,14]. Immunotherapy with IV corticosteroids led to a transient cessation of seizures in our case; however, the recurrence of breakthrough episodes and persistent cognitive deficits highlights the necessity for long-term immunological and neuropsychiatric management.

Autoantibodies targeting CASPR2 are associated with a spectrum of autoimmune neurological disorders. The most common clinical presentation is limbic encephalitis, characterized by subacute onset of memory impairment, confusion, mood disturbances, and seizures, often of temporal lobe origin [7,9,11,16,17]. According to a 10-year retrospective study on 53 CASPR2-IgG-seropositive patients at Mayo Clinic Neuroimmunology [17], the clinical phenotype of CASPR2 encephalitis typically encompasses a triad of CNS features, autonomic instability, and peripheral nerve hyperexcitability. These data also revealed that three (15%) CASPR2-IgG-seropositive patients with seizures exhibited coexisting PNS involvement. Of these, two patients demonstrated peripheral nerve hyperexcitability on electrodiagnostic studies, while one patient was diagnosed with small fiber neuropathy based on thermoregulatory sweat testing and autonomic reflex testing [17]. In our case, the first two (CNS features and autonomic instability) were predominant. Although the patient had a subjective feeling of generalized muscle twitching, the EMG/NCS did not find electrodiagnostic evidence of peripheral nerve hyperexcitability, including increased insertional activity, neuromyotonic discharges, myokymic discharges, and fasciculations.

Cognitive deficits (especially memory impairment) are one of the hallmark CNS features related to CASPR2-related autoimmune encephalitis, affecting >60% of patients [10,18]. In our case, the neuropsychological testing revealed mostly abnormal results in verbal learning and memory, along with a subtle vulnerability in semantic access efficiency. Boyko et al. (2020) [5] further expand the spectrum of CASPR2-associated disease to include limbic encephalitis, Morvan syndrome, and neuromyotonia, emphasizing that cognitive impairment, focal seizures, and emotional lability are common CNS manifestations. Their findings also report autonomic disturbances, such as hyperhidrosis, cardiac arrhythmias, and labile blood pressure, as frequent, which may elucidate our patient's intermittent sweating episodes and vasovagal-like events. Additional core features may include cerebellar symptoms, insomnia, neuropathic pain, and weight loss, underscoring the heterogeneous clinical presentation of the disease [11].

Seizures may be the initial or predominant symptom and can manifest in various forms, including focal seizures with impaired awareness, generalized tonic-clonic seizures, and FBDS. Although FBDS are more classically associated with LGI1 antibodies, they have also been reported in CASPR2-positive patients, suggesting phenotypic overlap and shared pathogenic mechanisms [8,9]. Overall, seizures in CASPR2 encephalitis are predominantly focal in nature, often with mesial temporal lobe involvement [19], as supported by EEG data in our case. In a multicenter retrospective study involving 25 patients with CASPR2 antibody-associated encephalitis, cognitive disturbance was the most frequent symptom, observed in 68% of cases. Limbic encephalitis was diagnosed in 32%, and seizures occurred in 24% of patients. Notably, CASPR2 antibodies were detected in both serum and CSF in a subset of patients, with dual positivity correlating with more severe disease and poorer initial response to immunotherapy [10]. Similarly, our patient required additional IVIG and corticosteroid therapy, in addition to anti-seizure medications, to achieve effective seizure control. MRI findings often reveal hyperintensities in the medial temporal lobes, while CSF analysis may show elevated protein levels or mild pleocytosis. EEG frequently demonstrates slow background activity and epileptiform discharges, particularly in the temporal regions, as observed in our case [10].

Additionally, recent studies have emphasized the importance of considering autoimmune etiologies in new-onset epilepsy, particularly in adults without prior seizure history. In a cohort of patients with autoimmune epilepsy, CASPR2 antibodies were identified in a subset presenting with isolated seizures, suggesting that autoimmune epilepsy may be underrecognized and misdiagnosed as idiopathic or structural epilepsy [15].

The pathogenesis of CASPR2 antibody-mediated encephalitis involves both humoral and cellular immune mechanisms. CASPR2 antibodies are predominantly of the IgG4 subclass, which is less efficient at activating complements but can disrupt protein-protein interactions and lead to internalization of surface antigens. In some cases, IgG1 antibodies are also present, potentially contributing to complement-mediated cytotoxicity and more aggressive disease [4]. The presence of CASPR2 antibodies in the CSF suggests intrathecal synthesis and direct CNS involvement, which may explain the severity of symptoms in these patients [9].

Recent systematic reviews [5,20] underscore the importance of early initiation of immunotherapy, with corticosteroids, IVIG, and plasma exchange serving as primary treatment modalities. In refractory cases, second-line agents such as rituximab or cyclophosphamide may be necessary. Clinical improvement is often observed within weeks of initiating therapy, although relapses can occur, necessitating long-term follow-up and maintenance immunosuppression in some patients [10]. A substantial subset of patients continues to experience residual cognitive and psychiatric symptoms despite aggressive treatment [5,11,19]. Similarly, our patient exhibited some residual memory deficits approximately four to five months after initiation of immunotherapy. Prognosis varies according to disease severity, antibody titers, and treatment response; however, many patients achieve substantial recovery with appropriate intervention [5]. Overall, this prognostic variability highlights the need for individualized therapeutic strategies and longitudinal follow-up to optimize outcomes and reduce long-term neurological morbidity.

## Conclusions

In conclusion, this case underscores the critical importance of maintaining a high index of suspicion for autoimmune epilepsy in patients presenting with unexplained new-onset focal seizures and systemic symptoms, such as cognitive decline, particularly in the absence of typical risk factors. It highlights the broad and variable clinical spectrum of CASPR2 antibody-associated disease and the necessity of early, sustained immunotherapy to reduce the risk of long-term neurological sequelae. Moreover, this case exemplifies the diagnostic and therapeutic challenges that healthcare providers may encounter when managing such complex presentations. Continued research is essential to improve recognition, refine treatment strategies, and optimize long-term outcomes in CASPR2 antibody-associated autoimmune epilepsy and encephalitis.
